# Coenzyme Q10 protects against burn‐induced mitochondrial dysfunction and impaired insulin signaling in mouse skeletal muscle

**DOI:** 10.1002/2211-5463.12580

**Published:** 2019-01-19

**Authors:** Harumasa Nakazawa, Kazuhiro Ikeda, Shohei Shinozaki, Shingo Yasuhara, Yong‐Ming Yu, J.A. Jeevendra Martyn, Ronald G. Tompkins, Tomoko Yorozu, Satoshi Inoue, Masao Kaneki

**Affiliations:** ^1^ Department of Anesthesia, Critical Care and Pain Medicine Massachusetts General Hospital Harvard Medical School Charlestown MA USA; ^2^ Shriners Hospitals for Children Boston MA USA; ^3^ Department of Anesthesiology Kyorin University School of Medicine Tokyo Japan; ^4^ Division of Gene Regulation and Signal Transduction Research Center for Genomic Medicine Saitama Medical University Japan; ^5^ Department of Life Sciences and Bioethics Tokyo Medical and Dental University Japan; ^6^ Department of Surgery Massachusetts General Hospital Harvard Medical School Boston MA USA; ^7^ Tokyo Metropolitan Institute of Gerontology Japan

**Keywords:** burn injury, coenzyme Q10, insulin resistance, mitochondrial dysfunction, skeletal muscle

## Abstract

Mitochondrial dysfunction is associated with metabolic alterations in various disease states, including major trauma (e.g., burn injury). Metabolic derangements, including muscle insulin resistance and hyperlactatemia, are a clinically significant complication of major trauma. Coenzyme Q10 (CoQ10) is an essential cofactor for mitochondrial electron transport, and its reduced form acts as a lipophilic antioxidant. Here, we report that burn injury induces impaired muscle insulin signaling, hyperlactatemia, mitochondrial dysfunction (as indicated by suppressed mitochondrial oxygen consumption rates), morphological alterations of the mitochondria (e. g., enlargement, and loss of cristae structure), mitochondrial oxidative stress, and disruption of mitochondrial integrity (as reflected by increased mitochondrial DNA levels in the cytosol and circulation). All of these alterations were significantly alleviated by CoQ10 treatment compared with vehicle alone. These findings indicate that CoQ10 treatment is efficacious in protecting against mitochondrial dysfunction and insulin resistance in skeletal muscle of burned mice. Our data highlight CoQ10 as a potential new strategy to prevent mitochondrial damage and metabolic dysfunction in burn patients.

**Abbreviations**
AA: antimycin A
ADP: adenosine diphosphate
Apo B: apolipoprotein B
ASC: apoptosis‐associated speck‐like protein containing a CARD
CLPP: caseinolytic mitochondrial matrix protease proteolytic subunit
CoQ10: coenzyme Q10
DAMP: damage‐associated molecular pattern
Drp1: dynamin‐related protein 1
FCCP: carbonyl cyanide 4‐(trifluoromethoxy) phenylhydrazone
Fis1: fission protein 1
GAPDH: glyceraldehyde 3‐phosphate dehydrogenase
GRP: glucose‐regulated protein
GSK: glycogen synthase kinase
HMGB1: high mobility group box 1
HSP: heat shock protein
IR: insulin receptor
IRS: insulin receptor substrate
LONP1: Lon protease 1
MFN: mitofusin
NADH: nicotinamide adenine dinucleotide
ND1: NADH dehydrogenase subunit 1
NLRP3: nucleotide‐binding domain leucine‐rich‐containing family pyrin domain‐containing‐3
OCR: oxygen consumption rate
Oligo: oligomycin
OPA1: optic atrophy 1
PINK1: PTEN‐induced putative kinase 1
ROS: reactive oxygen species
Rote: rotenone
SDHA: succinate dehydrogenase complex subunit A
Succ: succinate
TMPD: N,N,N9,N9‐tetramethyl‐p‐phenylenediamine
UPR: unfolded protein response

Mitochondrial dysfunction has been implicated in the pathogenesis of metabolic derangements and multi‐organ dysfunction after major trauma, including burn injury and sepsis. Metabolic alterations, which include insulin resistance 1, hyperlactatemia 2, hypermetabolism 3, and muscle wasting 4, are a major complication of burn injury and negatively affect the clinical outcome of burn patients. Previous studies have shown that a decrease in the activity of complex I, a major component of the mitochondrial electron transport chain in skeletal muscle, is associated with the severity of septic shock 5. Severe burn injury causes mitochondrial dysfunction in multiple organs, including skeletal muscle 6, 7. Therefore, improving mitochondrial function is thought to be a reasonable approach to ameliorating burn injury‐induced metabolic derangements and organ dysfunction.

Mitochondria are not just the power plants of cells, but lie at the crossroads of metabolism, apoptosis, and inflammation. When the integrity of the mitochondria is disrupted, mitochondrial DNA (mtDNA) is released from the mitochondria to the cytosol in which mtDNA activates nucleotide‐binding domain, leucine‐rich‐containing family pyrin domain‐containing‐3 (NLRP3) inflammasomes 8, 9. Moreover, when mtDNA is released to the circulation, mtDNA functions as a damage‐associated molecular pattern (DAMP) 10, inducing or enhancing systemic inflammation. Mitochondria are also a major source of reactive oxygen species (ROS) 11. Dysfunctional mitochondria often produce greater amounts of ROS, which, in turn, cause or exacerbate mitochondrial damage 12, 13.

Coenzyme Q10 is an essential cofactor for mitochondrial electron transport. It exists in oxidized (ubiquinone) and reduced (ubiquinol) forms. Reduced CoQ10 (ubiquinol) acts as a lipophilic antioxidant. Both forms are used as dietary supplements, but reduced CoQ10 (ubiquinol) has greater bioavailability compared to oxidized CoQ10 (ubiquinone) 14. Previous studies have shown that CoQ10 supplementation elicits beneficial effects in many human diseases, including heart failure 15, hypertension 16, and coronary artery disease 17, 18. It was reported previously that CoQ10 has the protective effects in rodent models of sepsis 19. An early study showed that exogenous CoQ10 treatment increases the CoQ10 content in the mitochondria 20. However, the effects of CoQ10 have not yet been studied in patients with, or animal models of, major trauma, including burn injury. Moreover, the effects of CoQ10 on function and/or morphology of the mitochondria have not yet been investigated in critical illness, including sepsis or major trauma.

We have previously shown that burn induces mitochondrial dysfunction and insulin resistance in skeletal muscle of mice 6, 21, 22, 23. We hypothesized that reduced form of CoQ10 (ubiquinol) has protective effects against burn injury‐induced mitochondrial dysfunction and insulin resistance in mouse skeletal muscle. To address this question, we examined the effects of CoQ10 in burned mice.

## Materials and methods

### Ethics statement

All experiments were carried out in accordance with the institutional guidelines, and the study protocol was approved by the Institutional Animal Care and Use Committee (IACUC) at Massachusetts General Hospital (protocol title: Stress‐Associated Insulin Resistance; protocol#: 2007N000020). The animal care facility is accredited by the Association for Assessment and Accreditation of Laboratory Animal Care.

### Animals

We used male CD1 mice (Charles River Laboratories, Boston, MA, USA) at 8 weeks of age. The mice were housed in a pathogen‐free animal facility with 12‐h light/dark cycles at 25 °C and fed with AIN‐93M (Research Diets, New Brunswick, NJ, USA). Burn injury was produced as described previously 6. Starting at 2 h after burn or sham‐burn, the mice were treated with reduced form of CoQ10 (ubiquinol) provided by Kaneka Nutrients Corp. (Pasadena, TX, USA) [40 mg·kg^−1^, subcutaneous injection (SC), b.i.d.] or vehicle (olive oil; Sigma, St. Louis, MO, USA) for 3 days. Three days after burn or sham‐burn, rectus abdominis muscle and plasma were collected for biochemical analyses. At the end of study, the mice were euthanized by carbon dioxide asphyxiation.

For detecting insulin signaling, at 3 days after burn or sham‐burn, following an overnight fasting, the mice received insulin (0.3 U·kg^−1^, Humulin R; Eli Lilly, Indianapolis, IN, USA) or saline via the portal vein under anesthesia with pentobarbital sodium (50 mg·kg^−1^, IP), and rectus abdominis muscles were collected at 5 min thereafter.

### Measurement of mitochondrial respiration

Seahorse XFp Extracellular Flux Analyzer (Seahorse Bioscience, North Billerica, MA, USA) was used to measure mitochondrial oxygen consumption rate (OCR) as an indicator of mitochondrial respiration. As described previously, we used fresh rectus abdominis muscle 6. Measurement of mitochondrial respiration was performed as described previously 6.

### Measurement of ATP content in skeletal muscle

At 3 days after burn or sham‐burn, fresh rectus muscle (50 mg) was homogenized in 1 mL of homogenization buffer B (0.25 m sucrose and 10 mm HEPES/NaOH, pH 7.4) for 30 s three times (30‐s interval) on ice. The homogenates were centrifuged at 1,000 g for 10 min at 4 °C. The supernatants (100 μL) were transferred to new tube, and 100 μL of ice‐cold 10% TCA was quickly added, followed by shaking for 20 s. After shaking, the mixtures were centrifuged at 10 000 g for 10 min at 4 °C. The supernatants (133 μL) were transferred to new tube, and 67 μL of neutralization buffer (1 m Tris‐acetate, pH 7.75) was added. The mixtures were diluted 30‐fold with deionized water, and ATP content was measured by commercial kit (ENLITEN ATP Assay Kit; Promega, Fitchburg, WI, USA) according to the manufacturer's instructions. The ATP content was normalized by protein concentration of homogenate.

### Electron microscopy

Electron microscopy was performed as described previously 6.

### Blue native PAGE

Two‐dimensional blue native PAGE was performed as described previously 24. The two‐dimensional SDS/PAGE and immunoblotting were performed according to standard protocols, and the blot was probed with anti‐NDUFA9 (#459100), anti‐succinate dehydrogenase complex subunit A (SDHA) (#459200) (Invitrogen, Waltham, MA, USA), and anti‐UQCRC2 (#ab14745) (Abcam, Cambridge, UK).

### Tissue homogenization and immunoblotting

At 3 days after burn or sham‐burn, following 4‐h fasting, rectus abdominis muscle was collected, homogenized, and blotted as described previously 6. The membranes were soaked in blocking buffer (GE Healthcare, Pittsburgh, PA, USA) for 1 h and then incubated overnight at 4 °C with anti‐glyceraldehyde 3‐phosphate dehydrogenase (GAPDH) (#2275‐PC‐1; Trevigen, Gaithersburg, MD, USA), anti‐Akt (#4691), anti‐phospho‐Akt at threonine 308 (#2965), anti‐phospho‐Akt at serine 473 (#4058), anti‐phospho‐Drp1 at serine 637 (#4867), anti‐phospho‐GSK‐3β at serine 9 (#9336), anti‐Parkin (#4216), anti‐HSP60 (#12165), anti‐HSP90 (#4877), anti‐GRP75 (#3593) (Cell Signaling Technology, Danvers, MA, USA), anti‐mitofusin‐1 (ab57602), anti‐mitofusin‐2 (ab56889), anti‐OPA1 (ab42364), anti‐RISP (#ab14746), anti‐UQCRC2 (ab14745), anti‐CLPP (ab124822) (Abcam), anti‐IR (07‐724), anti‐IRS1 (06‐248 (Millipore, Billerica, MA, USA), anti‐phospho‐IR (44‐800G), anti‐phospho‐IRS1 (44‐816G) (Life Technologies, Carlsbad, CA, USA), anti‐Drp1 (#611112), anti‐GSK‐3β (#610202) (BD Bioscience, San Jose, CA), anti‐OMA1 (17116‐1‐AP), anti‐YME1L1 (115101‐AP) (Proteintech, Chicago, IL), anti‐Fis1 (GTX111010; Genetex, Simpson, PA), Caspase‐1 (sc‐154; Santa Cruz Biotechnology, Santa Cruz, CA), anti‐IL‐1β (#AB401; R and D Systems, Minneapolis, MN), anti‐NDUFA9 (#459100), anti‐SDHA (#459200) (Invitrogen), anti‐Asc (AG‐25B‐0006), anti‐NLRP3 (AG‐20B‐0014) (Adipogen, San Diego, CA), and anti‐PINK1 (cc10006283; Cayman Chemical, Ann Arbor, CA), followed by incubation with HRP‐conjugated anti‐rabbit IgG or anti‐mouse IgG antibody (GE Healthcare) for 1 h at room temperature. Immunoreactive bands were detected with Lumigen ECL Ultra (Lumigen, Southfield, MI) and scanned using the HP Scanjet 4850 (Hewlett‐Packard, Palo Alto, CA). Densitometric analysis of the results was carried out using imagej software (ImageJ, Bethesda, MD, USA). Protein expression was normalized to GAPDH unless otherwise indicated.

### Mitochondria isolation and measurement of oxidized protein in mitochondria

Mitochondria were isolated from rectus abdominis using an adapted protocol. Briefly, the rectus abdominis was collected 3 days after burn or sham‐burn and homogenized in mitochondria isolation buffer (0.01% digitonin, 10 mm Tris/HCl, 0.25 mm sucrose, 10 mm KCl, 1.5 mm MgCl_2_, 10 mm EDTA, pH 7.4) with a Teflon pestle on glass. The homogenates were centrifuged at 500 ***g*** for 15 min. The supernatants were centrifuged at 10 000 ***g*** for 15 min. The mitochondrial pellets were resuspended and the centrifugation was repeated. The final pellets were resuspended in a minimal volume of mitochondria isolation buffer, and protein concentration was measured by the Bradford method.

One microgram of mitochondria fraction, isolated from rectus muscle, was applied into each well. Oxidized (carbonylated) protein in mitochondria was measured by commercial kit (Oxidized Protein Detection Kit, ab178020; Abcam) according to the manufacturer's instructions.

### Measurement of mtDNA‐to‐nuclear DNA ratio in skeletal muscle, and mtDNA content in cytoplasmic fraction and plasma

For measurement of mtDNA and nuclear DNA (nDNA) content in whole skeletal muscle, DNA was isolated from rectus muscle by proteinase K digestion. Rectus abdominis tissues (10 mg) were incubated in 200 μL of digestion mix (500 mU proteinase K, 20 mm Tris/HCl, 50 mm KCl, 10 mm EDTA, pH 8.4) at 55 °C for 3 days. After incubation at 95 °C for 1 h, the remains were spun down and the supernatants were diluted in 100 μL of TE buffer with 1 ng·μL^−1^ of salmon sperm DNA (Sigma).

For measurement of mtDNA content in cytoplasmic fraction in skeletal muscle, DNA in cytoplasmic fraction was collected by the following method. Firstly, fresh rectus abdominis muscle was homogenized in mitochondria isolation buffer (0.01% digitonin, 10 mm Tris/HCl, 0.25 mm sucrose, 10 mm KCl, 1.5 mm MgCl_2_, 10 mm EDTA, pH 7.4) with a Teflon pestle on glass on ice. The homogenates were centrifuged at 2300 rpm for 15 min. The supernatants were centrifuged at 9000 rpm for 15 min twice. Then, the supernatants were collected as cytoplasmic fraction. Secondly, the isolated cytoplasmic fractions (1 mg·mL^−1^, 100 μL) were incubated in 200 μL of digestion mix at 55 °C for 3 days. After incubation at 95 °C for 1 h, the remains were spun down and the supernatants were diluted in 100 μL of TE buffer with 1 ng·μL^−1^ of salmon sperm DNA.

For measurement of mtDNA in plasma, DNA was collected by commercial DNA extraction kit (Blood Mini Kit; Qiagen, Hilden, Germany), according to the manufacturer's instructions.

For evaluation of mtDNA, the NADH dehydrogenase subunit 1 (ND1) gene was amplified. The nuclear‐encoded apolipoprotein B (Apo B) gene was used for normalization. 1 μL of the diluted DNA was amplified in 12 μL of PCR mixture containing SYBR Green Master Mix (Life Technologies, Grand Island, NY, USA) and 1 μm of each primer. The amplification was measured by Mastercycler (Eppendorf, Westbury, NY). The primers were designed to target mtDNA (ND1) (Forward: 5′‐CAGGATGAGCCTCAAACTCC‐3′; Reverse: 5′‐GGTCAGGCTGGCAGAAGTAA‐3′) and nDNA (ApoB) (Forward: 5′‐CGTGGGCTCCAGCATTCTA‐3′; Reverse: 5′‐TCACCAGTCATTTCTGCCTTTG‐3′).

### Measurement of plasma lactate and HMGB1 concentrations

Concentrations of L‐lactate and high mobility group box 1 (HMGB1) in heparinized plasma were measured at 3 days after burn or sham‐burn using a commercial kit for L‐lactate (BioVision, Milpitas, CA, USA) and ELISA kit for HMGB1 (Shino‐Test Corporation, Tokyo, Japan), respectively, according to the manufacturers’ instructions.

### Isolation of total RNA and quantitative reverse transcription polymerase chain reaction

Total RNA was isolated with TRIzol reagent (Life Technologies) from skeletal muscle at 3 days after burn of sham‐burn injury. The first‐strand cDNA was synthesized from 1 μg of total RNA using the High Capacity cDNA Reverse Transcription Kit (Applied Biosystems, Waltham, MA, USA). RT‐PCR was performed using 10 ng of cDNA and TaqMan probes (Applied Biosystems) for IFN‐γ (Mm01168134_m1), IL‐1α (Mm00439620_m1), IL‐1β (Mm99999061_mH), TNF‐α (Mm00443258_m1), TLR4 (Mm00445273_m1), caspase‐11 (Mm01297328_m1), and 18S ribosomal RNA (Hs99999901_s1), conducted with Mastercycler ep realplex (Eppendorf, Hamburg, Germany). mRNA levels were normalized to those of 18S ribosomal RNA.

### Statistical analysis

To analyze the effects of CoQ10 in burned and sham‐burned mice, the data were compared with one‐way ANOVA followed by Tukey's multiple comparison test. To analyze the effects of CoQ10 and insulin in burned and sham‐burned mice, two‐way ANOVA followed by Tukey's multiple comparison test was used. A value of *P* < 0.05 was considered statistically significant. All values are expressed as means ± SEM.

## Results

### CoQ10 ameliorated burn injury‐induced mitochondrial dysfunction

**Figure 1:**
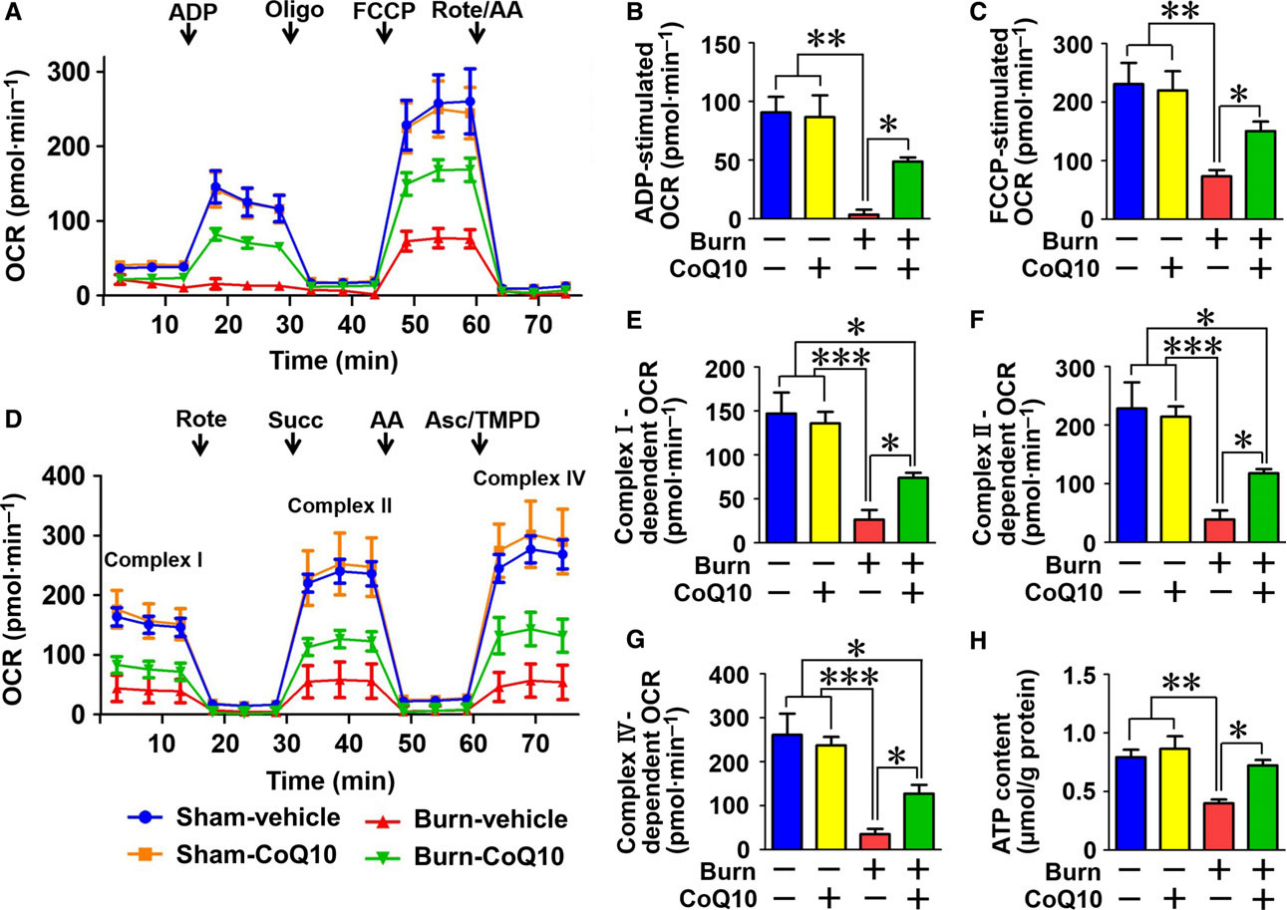
Coenzyme Q10 mitigated burn‐induced decreases in mitochondrial oxygen consumption rate (OCR) and ATP content in skeletal muscle. ADP‐ and FCCP‐stimulated OCRs were significantly decreased by burn injury compared with sham‐burn, both of which were ameliorated by CoQ10 treatment (A–C). Similarly, complex I‐, complex II‐, and complex IV‐dependent OCRs were significantly decreased by burn injury compared with sham‐burn, all of which were ameliorated by CoQ10 (D–G). ATP content in skeletal muscle was suppressed by burn injury compared with sham‐burn, which was mitigated by CoQ10 (H). ADP, adenosine diphosphate; Oligo, oligomycin; FCCP, carbonyl cyanide 4‐(trifluoromethoxy) phenylhydrazone; Rote, rotenone; AA, antimycin A; Succ, succinate; Asc, ascorbate; TMPD, N,N,N9,N9‐tetramethyl‐p‐phenylenediamine. The data were compared with one‐way ANOVA followed by Tukey's multiple comparison test. All values are presented as mean ± SEM. *P < 0.05, **P < 0.01, ***P < 0.001. *n* = 4 mice per group (A‐G). *n* = 6 mice per group (H).

To assess mitochondrial function, we evaluated the OCR in mitochondria isolated from skeletal muscle. Burn injury suppressed the ADP‐stimulated OCR and the FCCP‐stimulated OCR capacity 6. The latter was evaluated by measuring OCR stimulated by FCCP, a mitochondrial oxidative phosphorylation uncoupler. CoQ10 significantly ameliorated burn‐induced decreases in ADP‐stimulated and FCCP‐stimulated OCRs (Fig. 1A–C).

Next, we evaluated complex I‐, complex II‐, and complex IV‐dependent electron transfer capacity by measuring rotenone‐inhibitable, succinate‐stimulated, and ascorbate‐plus‐TMPD‐stimulated OCRs, respectively. Burn injury markedly suppressed complex I‐, complex II‐, and complex IV‐dependent OCRs in mouse skeletal muscle (Fig. 1D–H). CoQ10 mitigated the suppressed OCRs and altered ATP synthesis in burned mice (Fig. 1A–H). On the other hand, CoQ10 did not alter mitochondrial function in sham‐burned mice. Together, these results indicate that CoQ10 mitigated burn‐induced suppression of mitochondrial oxidative phosphorylation.

Consistent with our results of burn‐induced suppression of mitochondrial oxidative phosphorylation and its mitigation by CoQ10, burn injury decreased ATP content in skeletal muscle while CoQ10 prevented it (Fig. 1H). CoQ10 did not alter ATP content in sham‐burned mice.

### CoQ10 treatment prevented morphological alterations of the mitochondria in skeletal muscle

**Figure 2:**
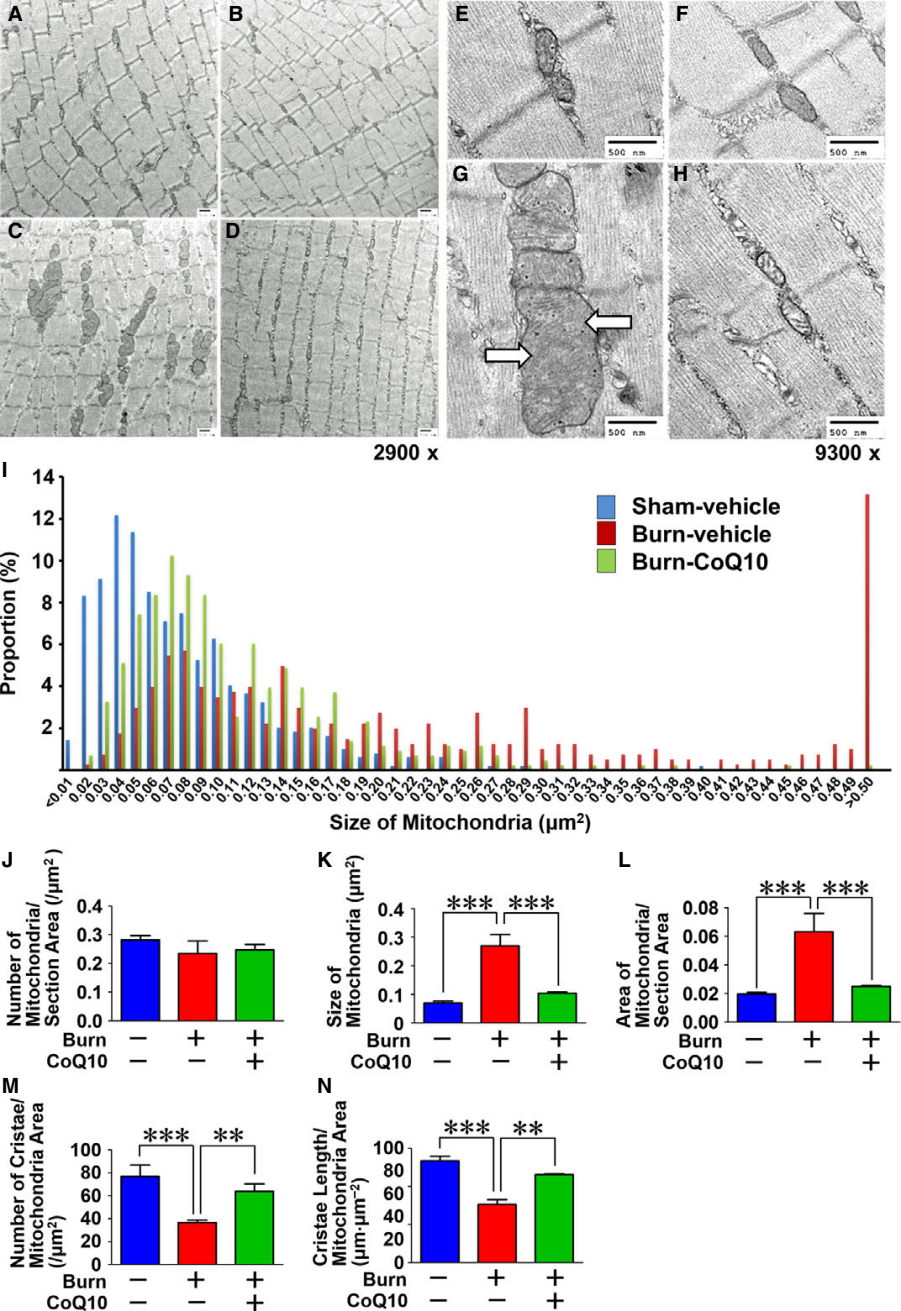
Coenzyme Q10 prevented burn injury‐induced morphological alterations of mitochondria in muse skeletal muscle. Burn injury induced the morphological alterations, enlargement (A–L) and loss of cristae structure (A–H, M, and N), in mitochondria, both of which were prevented by CoQ10. White arrows indicate loss of cristae structure (G). A, E: sham + vehicle; B, F: sham + CoQ10; C,G: burn + vehicle; D, H:burn + CoQ10. The data were compared with one‐way ANOVA followed by Tukey's multiple comparison test. All values are presented as mean ± SEM. **P < 0.01, ***P < 0.001. *n* = 3 mice per group.

Consistent with our recent study 6, burn injury induced morphological alterations in the intermyofibrillar mitochondria, namely the enlargement and loss of cristae structure (Fig. 2). While the number of mitochondria was not significantly altered by burn injury (Fig. 2J), the size of the individual organelles and total surface area of mitochondria were increased subsequent to burn, whereas CoQ10 treatment significantly inhibited the enlargement of mitochondria induced by burn injury, as evidenced by the reversal of these effects by CoQ10 (Fig. 2K,L). Similarly, CoQ10 treatment prevented burn injury‐induced loss of cristae structure, which was assessed by measuring the burn‐induced decrease in number and length of cristae and the reversal thereof by CoQ10 (Fig. 2M,N).

### CoQ10 ameliorated burn‐induced decrease in mitochondrial supercomplex assembly in mouse skeletal muscle

**Figure 3:**
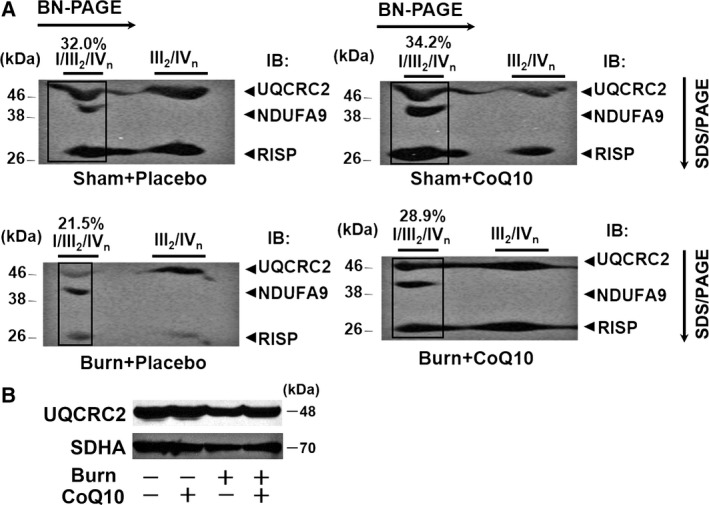
Coenzyme Q10 prevented burn‐induced impaired mitochondrial respiratory supercomplex assembly in skeletal muscle. Respiratory supercomplexes in the mitochondria were separated by blue native PAGE followed by two‐dimensional SDS/PAGE and visualized using antibodies for NDUFA9 (a component of complex I) and UQCRC2 and RISP (components of complex III). The percentage of the complex I‐containing largest supercomplex (I/III_2_/IV_n_) signal intensity was decreased in vehicle‐treated burned mice compared with sham‐burned mice (A). CoQ10 inhibited the burn‐induced alteration in the supercomplexes. On the other hand, total protein abundance of UQCRC2 was not altered by burn injury or FTI‐277 (B). SDHA (a component of complex II) was used as a control. IB: immunoblotting.

Mitochondrial respiratory supercomplexes, which are composed of complex I, complex III, and complex IV, are important for efficient electron transport and oxidative phosphorylation 25, 26. Our previous study showed that burn injury decreased the mitochondrial supercomplex assembly in mouse skeletal muscle 6. Therefore, we assessed the effects of CoQ10 treatment on respiratory supercomplexes in the mitochondria of mouse skeletal muscle by two‐dimensional blue native PAGE (2D‐BN‐PAGE). As expected, multiple respiratory supercomplexes were detected in mouse skeletal muscle, including those consisting of (1) complex I, complex III, and complex IV; and (2) complex III and complex IV. 2D‐BN‐PAGE showed that when treated with vehicle alone, burn injury decreased these respiratory supercomplexes while CoQ10 restored the formation of respiratory supercomplexes in burned mice (Fig. 3A). All the respiratory supercomplexes included complex III. The total protein abundance of UQCRC2, a component of complex III, was not altered by burn injury or CoQ10 (Fig. 3B).

### Effects of burn injury and CoQ10 treatment on proteins involved in mitochondrial fusion and fission

**Figure 4:**
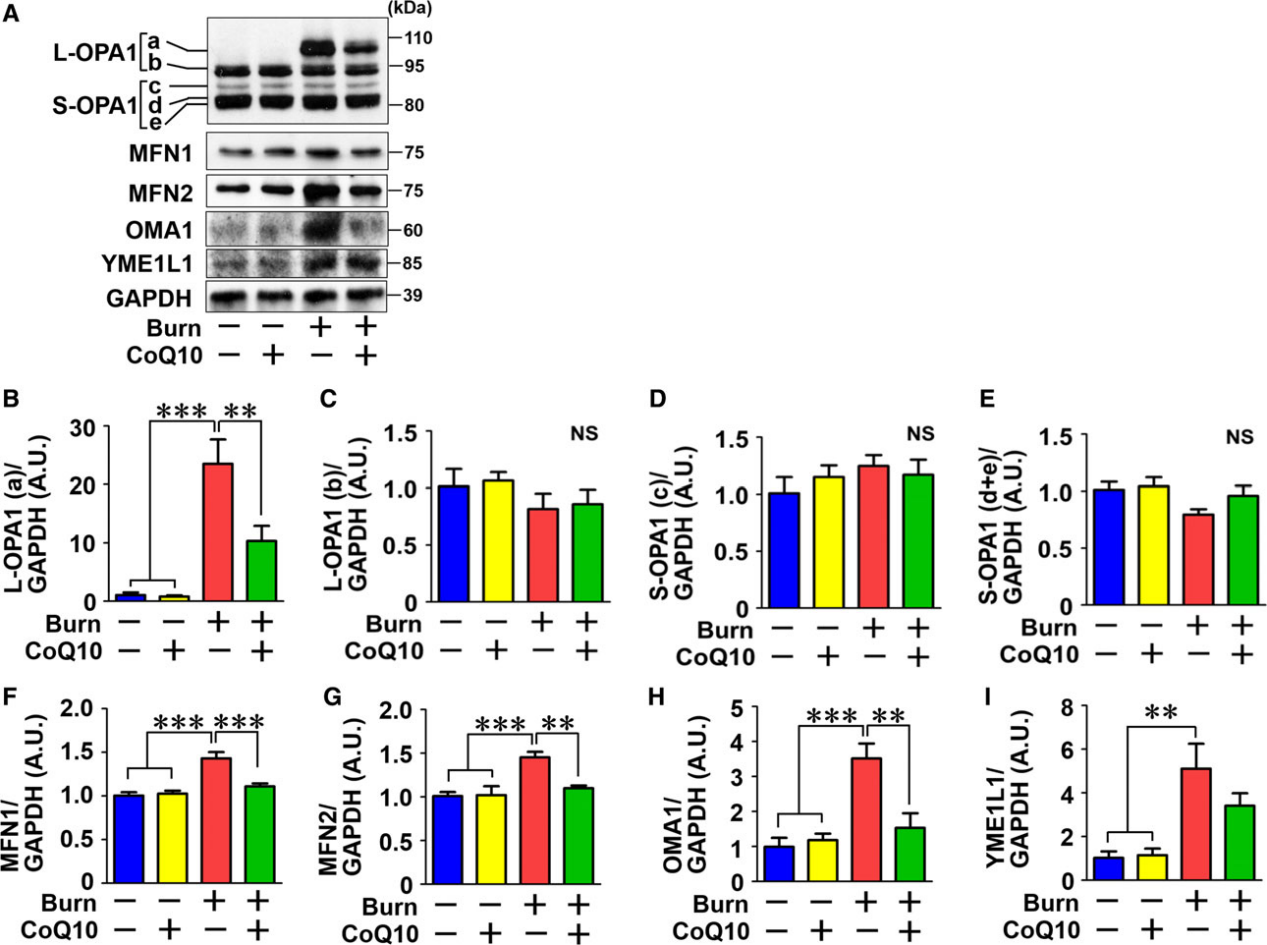
Effects of burn injury and CoQ10 on proteins involved in mitochondrial fusion in mouse skeletal muscle. The longest form of OPA1 (a) was induced by burn injury, which was ameliorated by CoQ10 (A, B). Protein expression of other isoforms (b–e) was not altered by burn injury or CoQ10 (A, C–E). MFN1, MFN2, OMA1, and YME1L1 protein expression was increased by burn injury (A, F–I). CoQ10 significantly decreased MFN1, MFN2, and OMA1 expression in burned mice. CoQ10 appears to decrease YME1L1 expression in burned mice, but there was no statistical difference. The data were compared with one‐way ANOVA followed by Tukey's multiple comparison test. All values are presented as mean ± SEM. **P < 0.01, ***P < 0.001, NS: not significant. *n* = 6 mice per group.

The morphological alterations of mitochondria, particularly its enlargement, in burned mice suggest that both burn injury and CoQ10 alter mitochondrial dynamics, such as fission and fusion. Therefore, we evaluated the effects of burn injury and CoQ10 on proteins that regulate these properties. Burn injury significantly increased protein expression of mitofusin‐1 (MFN1), MFN2, and the long form of optic atrophy 1 (L‐OPA1) (Fig. 4A–C,F, and G), all of which play crucial roles in mitochondrial fusion 27, 28, 29. CoQ10 treatment significantly decreased expression of MFN1, MFN2, and L‐OPA1 in burned mice (Fig. 4B,F, and G). In contrast to the long form of OPA1, expression of the short form (S‐OPA1) was not altered by burn injury or CoQ10 (Fig. 4D,E). L‐OPA1 is cleaved by proteases OMA1 and YME1L1, which convert it to S‐OPA1. Loss of these proteases results in the accumulation of L‐OPA1 and concomitant decrease of S‐OPA1 30. We therefore examined whether burn injury decreased the expression of these proteases. Contrary to expectations, burn injury significantly increased OMA1 and YME1L1 expression (Fig. 4H,I), while CoQ10 treatment significantly decreased OMA1 expression in burned mice (Fig. 4H). Although the burn‐induced increase in YME1L1 expression appeared to be decreased by CoQ10 treatment, there was no statistical difference (Fig. 4I).

**Figure 5:**
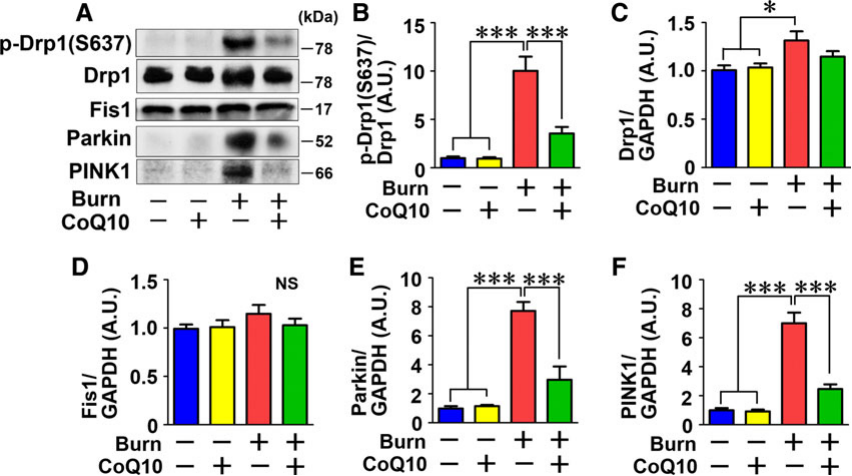
Effects of burn injury and CoQ10 on proteins involved in mitochondrial fission in mouse skeletal muscle. Burn injury increased phosphorylated Drp1 (p‐Drp1), which was inhibited by CoQ10 (A, B). Burn injury increased Drp1 protein expression compared with sham‐burn (A, C). When treated with CoQ10, Drp1 expression was no longer increased relative to sham‐burned mice. Neither burn injury nor CoQ10 altered Fis1 expression (A, D). Burn injury increased Parkin and PINK1 protein expression, which was ameliorated by CoQ10 (A, E, and F). The data were compared with one‐way ANOVA followed by Tukey's multiple comparison test. All values are presented as mean ± SEM. *P < 0.05, ***P < 0.001, NS, not significant. *n* = 6 mice per group.

Next, we evaluated expression of GTPase dynamin‐related protein 1 (Drp1) and mitochondrial fission protein 1 (Fis1), both of which promote mitochondrial fission 31. Burn injury significantly increased Drp1 protein expression, which was prevented by CoQ10 treatment (Fig. 5A and C). Phosphorylation of Drp1 at serine 637 inhibits the fission‐inducing activity of Drp1 32. Burn injury markedly increased phosphorylation of Drp1, which was prevented by CoQ10 treatment (Fig. 5A,B). Fis1 has important roles in mitochondrial fission by recruiting Drp1, and it is known to increase during fission 31. Neither burn injury nor CoQ10 altered Fis1 expression (Fig. 5A and D). Parkin and PTEN‐induced putative kinase 1 (PINK1) act as sensors for mitochondrial quality and homeostasis and are activated by mitochondrial damage 33. Burn injury markedly increased Parkin and PINK1 expression, which was prevented by CoQ10 (Fig. 5A, E, and F).

### CoQ10 supplementation inhibited burn injury‐induced mitochondrial oxidative stress

**Figure 6:**
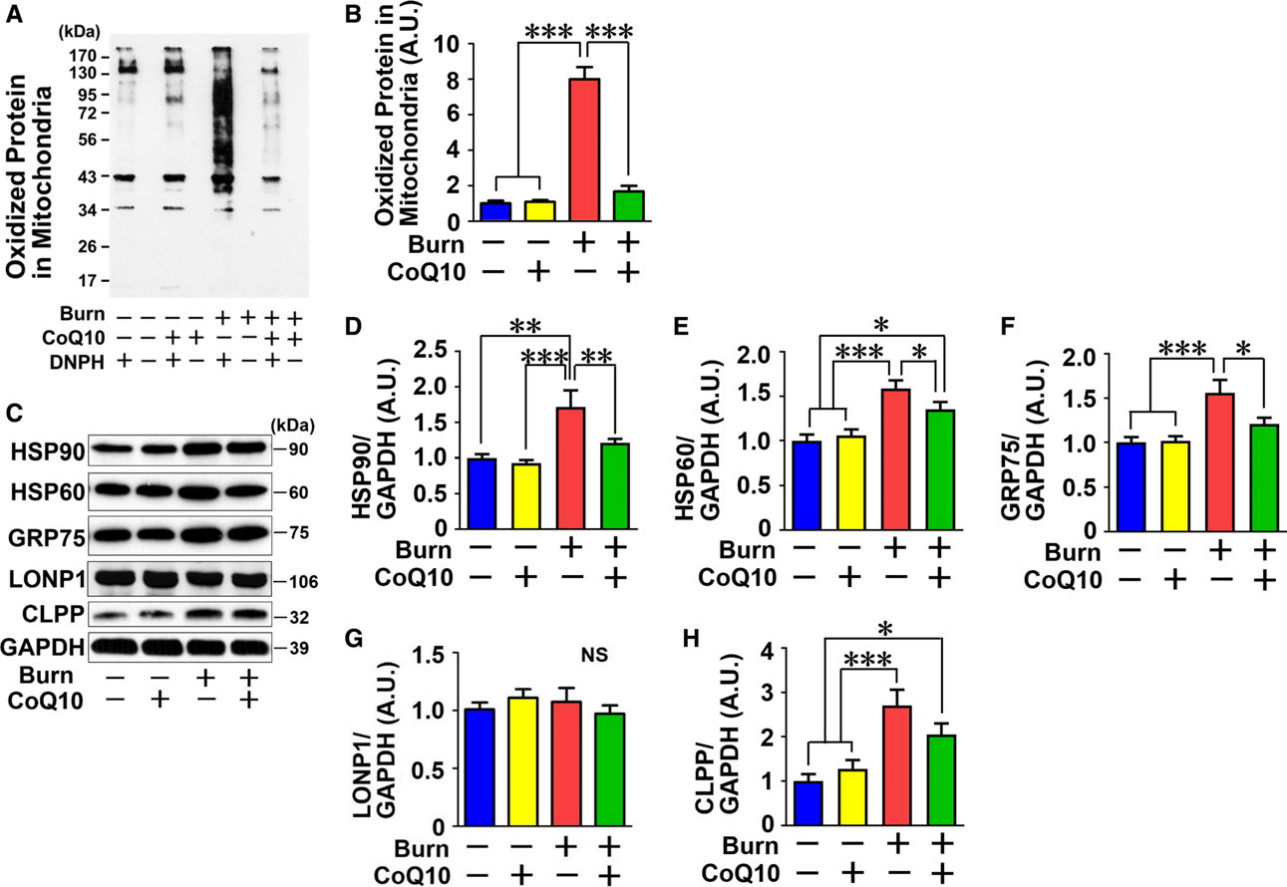
Coenzyme Q10 attenuated burn injury‐induced mitochondrial oxidative stress and mitochondrial unfolded protein response (mtUPR) in mouse skeletal muscle. To assess mitochondrial oxidative stress, oxidized (carbonylated) proteins in the mitochondria were measured. Burn injury increased oxidized proteins in the mitochondria, which was prevented by CoQ10 (A and B). Oxidized (carbonylated) proteins were detected only when incubated with DNPH, confirming the specificity. To assess mtUPR, protein expression of HSP90, HSP60, GRP75, and CLPP was measured. These protein expressions in skeletal muscle were significantly increased by burn injury, which were ameliorated by CoQ10 (C–F, H). LONP1 expression was not altered by burn and CoQ10 (C and G). DNPH: 2,4‐dinitrophenylhydrazine. The data were compared with one‐way ANOVA followed by Tukey's multiple comparison test. All values are presented as mean ± SEM. *P < 0.05, **P < 0.01, ***P < 0.001. *n* = 6 mice per group.

To assess oxidative stress in the mitochondria, we evaluated carbonylated proteins. Burn injury markedly increased carbonylated proteins in the mitochondria, and CoQ10 treatment prevented the burn‐induced increase in mitochondrial carbonylated proteins (Fig. 6A,B).

### CoQ10 supplementation attenuated burn injury‐induced mtUPR

The mitochondrial unfolded protein response (mtUPR)‐related protein levels are linked to mitochondrial stress levels. Therefore, we evaluated the effects of CoQ10 on mtUPR‐related proteins, such as heat shock protein (HSP) 60, HSP90, and glucose‐regulated protein (GRP) 75 (also known as mtHSP70) (Fig. 6C–F). Burn injury significantly increased protein expression of HSP60, HSP90, and GRP75, chaperone proteins 34, CoQ10 treatment significantly suppressed the induction of these proteins. ATP‐dependent caseinolytic mitochondrial matrix protease proteolytic subunit (CLPP) and Lon protease 1, mitochondrial (LONP1) are mitochondrial proteases, which are increased under mitochondrial stress, and they degrade unfolded proteins 35. In this study, CLPP was increased by burn injury, and CoQ10 appeared to attenuate the expression level of CLPP in burned mice, but it was not statistically significant (Fig. 6H); however, LONP1 was not changed by burn injury or CoQ10 treatment in this experiment (Fig. 6G).

### CoQ10 prevented burn‐induced alteration in mtDNA levels and inflammatory response

**Figure 7:**
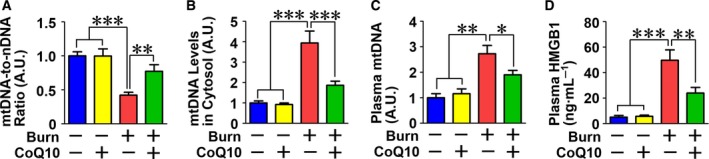
Coenzyme Q10 inhibited the effects of burn injury on mtDNA levels and circulating HMGB1. Burn injury decreased mtDNA‐to‐nDNA ratio, an indicator of total mtDNA content, compared with sham‐burn, which was mitigated by CoQ10 (A). In contrast, burn injury increased mtDNA levels in the cytosol (B) and plasma (C), both of which were ameliorated by CoQ10. Similar to plasma mtDNA, plasma HMGB1 concentration was increased by burn injury, which was inhibited by CoQ10 (D). The data were compared with one‐way ANOVA followed by Tukey's multiple comparison test. All values are presented as mean ± SEM. *P < 0.05, **P < 0.01, ***P < 0.001. *n* = 6 mice per group.

Mitochondrial dysfunction is often associated with a decrease in mtDNA content in many disease states, including LPS challenge 36. Burn injury decreased the mtDNA‐to‐nDNA ratio, which was prevented by CoQ10 (Fig. 7A). When the integrity of the mitochondria is disrupted, mtDNA is released from the mitochondria to the cytosol and circulation 37, 38 and functions as a mitochondrial DAMP. Burn injury increased mtDNA content in the cytosol compared with sham‐burn, which was inhibited by CoQ10 (Fig. 7B). Moreover, burn injury increased the plasma level of mtDNA, which was ameliorated by CoQ10 (Fig. 7C). Similarly, plasma HMGB1, a nonmitochondrial DAMP 39, was increased by burn injury, and CoQ10 decreased plasma HMGB1 in burned mice (Fig. 7D).

**Figure 8:**
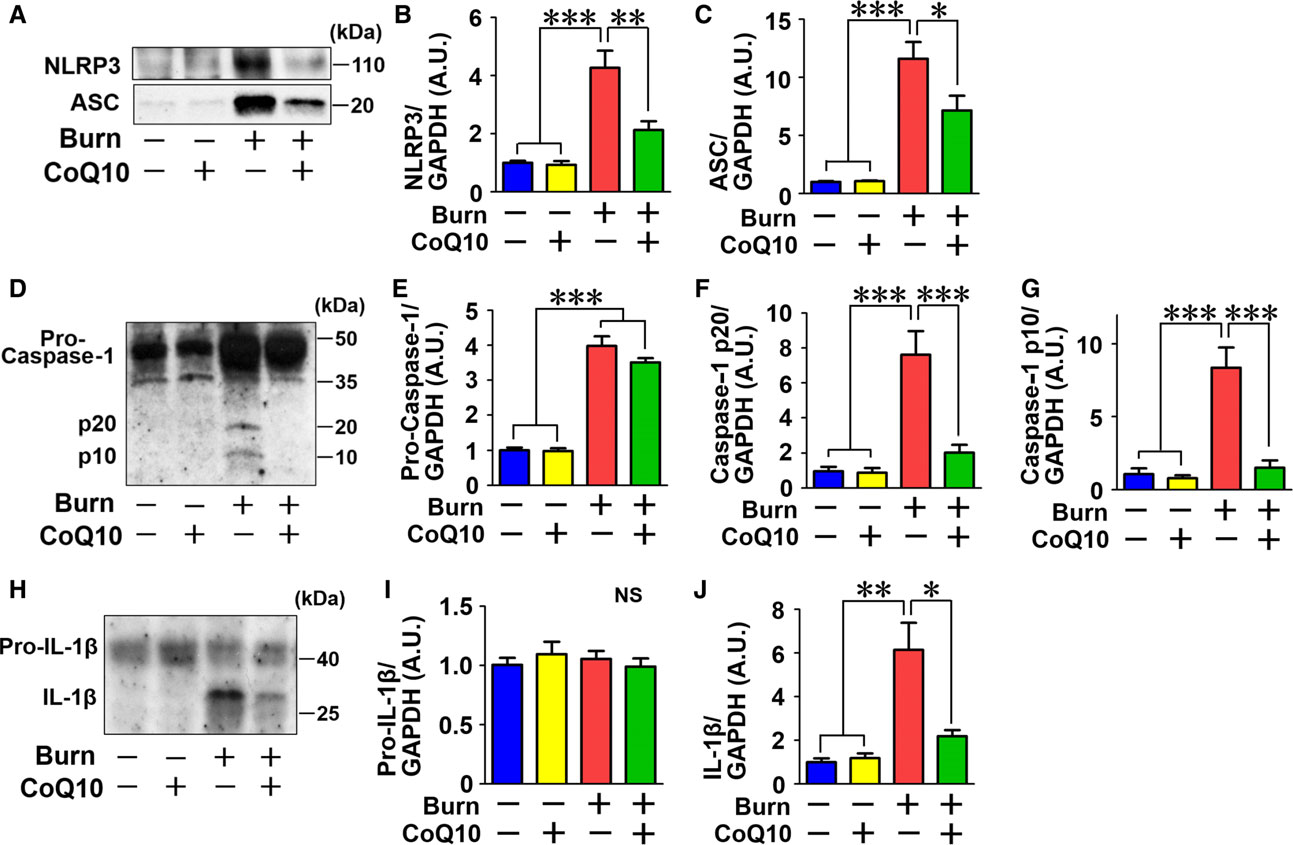
Coenzyme Q10 inhibited burn injury‐induced NLRP3 inflammasome activation in skeletal muscle. Burn increased protein expression of ASC and NLRP3, components of NLRP3 inflammasome (A–C). CoQ10 inhibited burn‐induced expression of ASC and NLRP3. Burn injury increased expression of pro‐caspase‐1 and cleaved caspase‐1 (p10 and p20) compared with sham‐burn (D–G). CoQ10 did not significantly decrease pro‐caspase‐1 expression, but inhibited burn injury‐induced expression of cleaved caspase‐1 (p10 and p20). Similarly, burn injury increased cleaved IL‐1β, which was ameliorated by CoQ10 (H–J). The data were compared with one‐way ANOVA followed by Tukey's multiple comparison test. All values are presented as mean ± SEM. *P < 0.05, **P < 0.01, ***P < 0.001. *n* = 6 mice per group.

**Figure 9:**
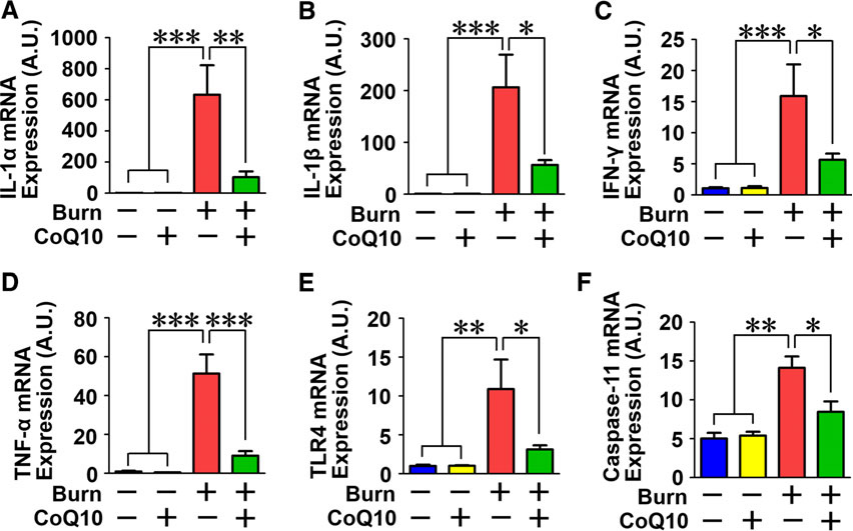
Coenzyme Q10 inhibited burn injury‐induced proinflammatory expression in skeletal muscle. Burn injury increased the mRNA levels of IL‐1α (A), IL‐1β (B), IFN‐γ (C), TNF‐α (D), TLR4 (E), and caspase‐11 (F), which were inhibited by CoQ10. The data were compared with one‐way ANOVA followed by Tukey's multiple comparison test. All values are presented as mean ± SEM. *P < 0.05, **P < 0.01, ***P < 0.001. *n* = 6 mice per group.

In the cytosol, mtDNA activates NLRP3 inflammasome 8, 9. We therefore evaluated the activation status of NLRP3 inflammasome. NLRP3 inflammasome activation induces cleavage (activation) of pro‐caspase‐1. Cleaved (activated) caspase‐1, in turn, cleaves pro‐IL‐1β and produces mature, active IL‐1β. NLRP3 inflammasome activation is associated with increased protein expression of the major components of NLRP3 inflammasome, including apoptosis‐associated speck‐like protein containing a CARD (ASC) and NLRP3 40. Burn injury markedly increased ASC and NLRP3 protein expression. CoQ10 significantly ameliorated burn‐induced increases in ASC and NLRP3 expression (Fig. 8A–C). Burn injury induced cleavage of p10 and p20 caspase‐1 which was prevented by CoQ10 (Fig. 8D–G). Burn injury increased pro‐caspase‐1 expression, but CoQ10 did not significantly decrease pro‐caspase‐1 expression in sham‐burned and burned mice (Fig. 8D,E). Consistently, burn injury induced cleavage of IL‐1β, whereas CoQ10 significantly inhibited burn‐induced cleavage of IL‐1β (Fig. 8H–J). Moreover, in line with the prevention of inflammatory responses by CoQ10, burn injury increased mRNA expression of proinflammatory genes IL‐1α, IL‐1β, IFN‐γ, and TLR4, while caspase‐11 was significantly inhibited by CoQ10 treatment in skeletal muscle (Fig. 9).

### CoQ10 prevented burn injury‐induced impaired insulin signaling in mouse skeletal muscle

**Figure 10:**
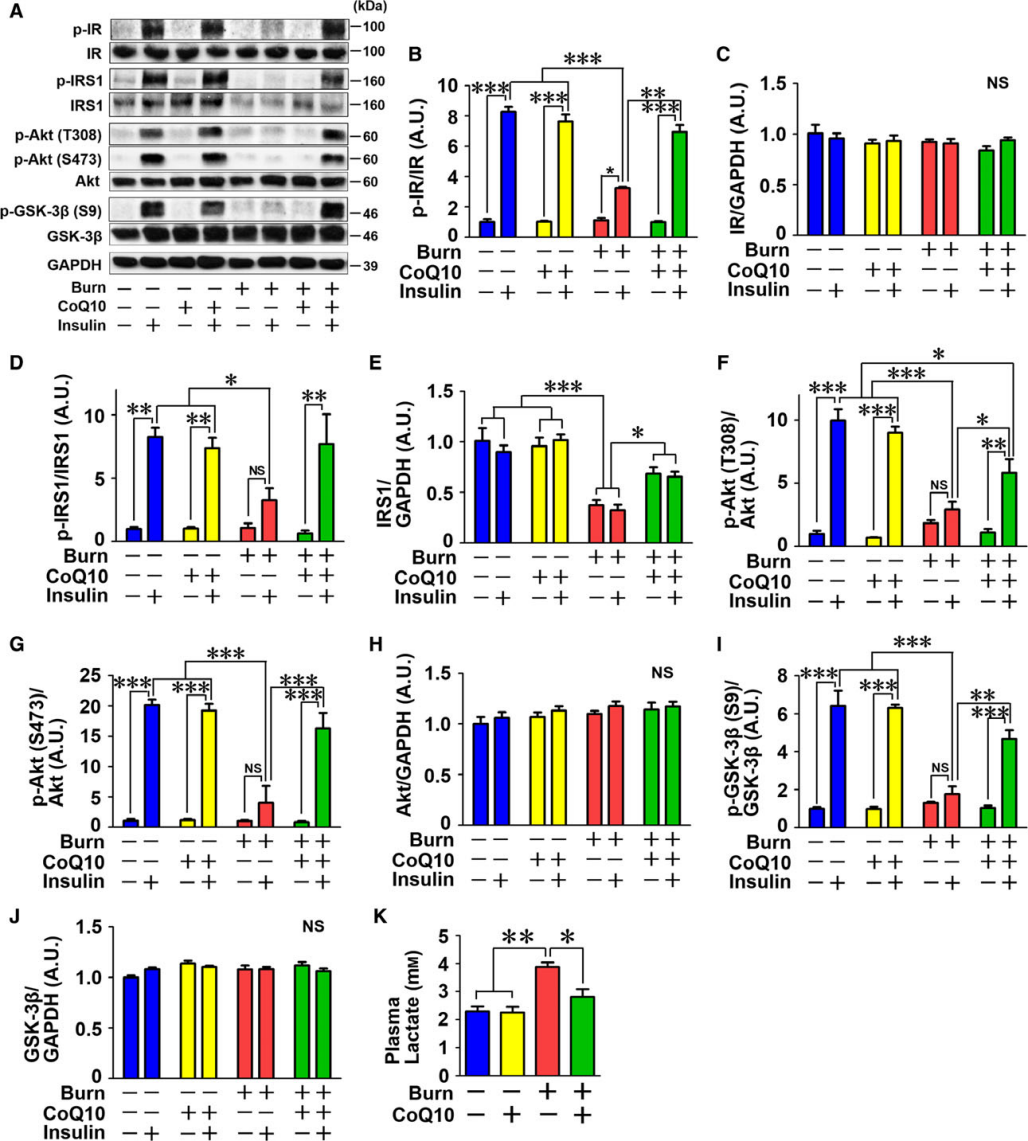
Coenzyme Q10 prevented burn injury‐induced impaired insulin signaling and hyperlactatemia in mouse skeletal muscle. Burn injury inhibited insulin‐stimulated phosphorylation of IR (B), IRS1 (D), Akt (F and G), and GSK‐3β (I) at 3 days after burn injury compared with sham‐burn. CoQ10 significantly ameliorated insulin‐stimulated phosphorylation of IR (B), IRS1 (D), Akt (F and G), and GSK‐3β (I) in burned mice. Protein expression of IRS1 was suppressed by burn injury, and CoQ10 increased IRS1 expression in burned mice (E). On the other hand, protein expression of IR (C), Akt (H), and GSK‐3β (J) was not altered by burn injury or CoQ10. Plasma lactate level was significantly increased at 3 days after burn injury, and CoQ10 supplementation ameliorated burn‐induced hyperlactatemia (K). The data were compared with two‐way ANOVA followed by Tukey's multiple comparison test. All values are presented as mean ± SEM. *P < 0.05, **P < 0.01, ***P < 0.001, NS: not significant. *n* = 4 mice per group (A–J), *n* = 6 mice per group (K).

Consistent with previous studies 22, 23, burn injury impaired insulin signaling in mouse skeletal muscle compared with sham‐burned mice at 3 days after burn injury (Fig. 10). The insulin receptor (IR)–insulin receptor substrate (IRS)‐1–Akt pathway plays a central role in the metabolic actions of insulin in skeletal muscle 41, 42. Insulin‐stimulated phosphorylation of IR was significantly decreased in burned mice compared with sham‐burned mice (Fig. 10A,B). IR protein expression was not altered by burn injury (Fig. 10A and C). On the other hand, burn injury significantly decreased IRS1 protein expression relative to sham‐burn (Fig. 10A and E). Insulin‐stimulated phosphorylation of IRS1 protein expression was also significantly decreased after burn injury (Fig. 10A and D). These burn injury‐induced alterations were significantly mitigated by CoQ10 supplementation (Fig. 10A,B,D, and E).

Next, we tested the effects of burn injury and CoQ10 on phosphorylation of the downstream insulin signaling molecules, Akt and glycogen synthase kinase (GSK)‐3β. Insulin injection markedly increased phosphorylation of Akt at threonine 308 and serine 473 and GSK‐3β at serine 9 in sham‐burned mice. In burned mice, however, insulin failed to significantly increase phosphorylation of Akt and GSK‐3β. CoQ10 supplementation significantly ameliorated burn injury‐induced suppression of insulin‐stimulated phosphorylation of Akt and GSK‐3β (Fig. 10A,F,G, and I). Protein expression of Akt and GSK‐3β was not altered by burn injury or CoQ10 (Fig. 1A,H, and J). On the other hand, CoQ10 treatment did not alter insulin signaling in sham‐burned mice.

### CoQ10 prevented burn‐induced hyperlactatemia

Consistent with our previous study, burn injury induced hyperlactatemia at 3 days after burn injury 22. CoQ10 treatment prevented burn injury‐induced hyperlactatemia compared with vehicle alone (Fig. 10K). CoQ10 did not alter plasma lactate levels in sham‐burned mice.

## Discussion

Here, we show that treatment with the reduced form of CoQ10 (ubiquinol) substantially reverses burn injury‐induced metabolic alterations (e.g., insulin resistance and hyperlactatemia), dysfunction (i.e., decreased mitochondrial OCRs), and oxidative stress in mouse skeletal muscle. In parallel, CoQ10 inhibits NLRP3 inflammasome activation and systemic inflammatory response after burn injury. Collectively, our data suggest that burn injury causes mitochondrial dysfunction, which thereby contributes to the metabolic dysfunction and inflammatory response, all of which are prevented by CoQ10. These results highlight a cause–effect relation between mitochondrial dysfunction and the metabolic aberration and inflammatory response associated with severe burn injury.

Mitochondrial dysfunction after burn injury is associated with gross morphological changes, namely mitochondrial enlargement and loss of cristae structure, in addition to inhibition of respiratory supercomplex assembly. Loss of cristae structure is linked to the disruption of respiratory supercomplex formation 43. When respiratory supercomplex formation is inhibited, it decreases the efficiency of oxidative phosphorylation in mitochondrial electron transport 43 and increases the generation of ROS, particularly in complex I 44. The observed effects on mitochondrial dysfunction and oxidative stress, and its prevention by CoQ10, correlate with the morphological alterations in burned mice. CoQ10 prevented these morphological alterations, promoted supercomplex assembly, and inhibited mitochondrial oxidative stress, in parallel with the mitigation of mitochondrial dysfunction as indicated by the increased OCRs associated with electron transport after CoQ10 administration. In addition, we clearly showed that CoQ10 suppressed burn‐induced increasing oxidized protein levels and mtUPR‐related proteins. These results indicate that CoQ10 ameliorates burn‐induced mitochondrial stress. Therefore, administration of CoQ10 attenuates the mitochondrial stress after burn injury and it shows that CoQ10 works to ameliorate mitochondrial stress.

The appearance of enlarged mitochondria was prominent after burn injury and was almost completely prevented by CoQ10. Of note, a previous study has shown that neurotoxin‐induced cognitive impairment and neurodegeneration are accompanied by the appearance of enlarged mitochondria with distorted cristae in rat hippocampus, which can be reversed by CoQ10 administration 45. In addition, congenital CoQ10 deficiency, which is caused by genetic disruption of *CoQ7,* results in embryonic lethality, where enlarged mitochondria are observed in mice 46. These findings raise the possibility that CoQ10 may play a protective role against the enlargement of mitochondria. Further studies are required to clarify this point. Mitochondrial enlargement was accompanied by increased expression of the proteins involved in mitochondrial fusion, namely MFN1, MFN2, and the long form of OPA1 (L‐OPA1). YME1L1 and OMA1 proteases cleave L‐OPA1 converting it to the short form (S‐OPA1). Loss of YME1L1 and OMA1 results in an increase in L‐OPA1 and a decrease in S‐OPA1. However, while L‐OPA1 was increased after burn injury, S‐OPA1 was not decreased. The protein expression of YME1L1 and OMA1 increased after burn injury. Together, the suppression of these proteases, YME1L1 and OMA1, is unlikely to play a role in the increase of L‐OPA1 after burn injury. On the other hand, the protein expression of Fis1, which plays an important role in fission, was not altered by burn injury or CoQ10. Burn injury increased the inhibitory phosphorylation of Drp1, which inhibits Drp1‐mediated fission. These data suggest that the relative increase in fusion over fission may contribute to the appearance of enlarged mitochondria. Other mechanism(s), however, may also underlie the mitochondrial morphological alterations after burn injury.

Burn injury induced the depletion of mtDNA as demonstrated by the decrease of the mtDNA‐to‐nDNA ratio in skeletal muscle. On the other hand, mtDNA increased after burn injury in both the cytosolic fraction and circulation. When mtDNA is released from the mitochondria, it activates NLRP3 inflammasome within cells 8, 9 whereas it functions as a DAMP in the circulation 10. Our data show that CoQ10 prevents mtDNA release from the mitochondria to the cytosol and the circulation. These data indicate that CoQ10 mitigates mitochondrial dysfunction and thereby inhibits NLRP3 inflammasome activation and systemic inflammatory response after burn injury. Consistent with the mitigation of systemic inflammatory response, CoQ10 decreased plasma HMGB1 levels in burned mice. Inflammatory responses, such as NLRP3 inflammasome, play a critical role in the pathogenesis of insulin resistance 47. Impaired insulin signaling is a hallmark of insulin resistance. It is conceivable that CoQ10 acts to lessen the severity of impaired insulin signaling, at least in part, by preventing inflammation. Together, our data suggest that mitigation of burn‐induced mitochondrial dysfunction by CoQ10 might contribute to the mollification of inflammatory response and metabolic dysfunction.

Hyperlactatemia is a predicator of the mortality of burn patients 2. A previous study has shown that over and above impaired microcirculation, which causes a resultant deficit in oxygen availability in tissues, metabolic alteration contributes to hyperlactatemia in burn patients 3. Skeletal muscle is a major organ that produces and secretes lactate into the circulation. Mitochondrial dysfunction exacerbates lactate production by increasing aerobic glycolysis in skeletal muscle. In addition, muscle‐specific gene disruption of insulin receptor substrate (IRS)1 and IRS2 causes hyperlactatemia, but does not increase blood glucose levels in mice 48. Therefore, it is possible that impaired insulin signaling could contribute to hyperlactatemia. Farther more, the shift of mitochondrial respiration to glycogenic ATP synthesis, the Warburg‐like effect, plays an important role in hyperlactatemia after burn injury.

This study shows that CoQ10 can prevent mitochondrial dysfunction and mitochondrial oxidative stress, while mitigating metabolic derangements and local and systemic inflammatory response in skeletal muscle of mice. Our data highlight the potential utility of the reduced form of CoQ10 (ubiquinol) as a novel therapeutic strategy for improving the clinical outcome of severely burn patients.

## Conflict of interest

The authors declare no conflict of interest.

## Author contributions

MK and HN conceptualized and coordinated the project. HN and KI acquired the data. HN and KI analyzed and interpreted the data. MK, TY, and SI supervised the project. HN, SS, and SY validated the data. MK, SS, and HN wrote original draft. MK, HN, KI, SS, SI, and RGT provided funding to support the research; and MK, HN, KI, SS, SY, Y‐MY, JAJM, RGT, TY, and SI reviewed and edited the manuscript.
